# Delayed Diagnosis of a Large Parietal Meningioma Presenting as Progressive Proprioceptive Loss: A Case Report and Review of Diagnostic Bias Across Specialties

**DOI:** 10.7759/cureus.110967

**Published:** 2026-06-16

**Authors:** Mahdi N Fadel, Ali Fadel, Farah Fadel, Marwan Shuayto

**Affiliations:** 1 Medicine, American University of the Caribbean School of Medicine, Cupecoy, SXM; 2 Neurology, Henry Ford Providence Southfield Hospital, Southfield, USA; 3 Research, Wayne State University, Detriot, USA; 4 Pharmacology, CVS Health, Lansing, USA; 5 Neurology, Michigan Neurology and Spine Center, Port Huron, USA

**Keywords:** bias in medicine, brain meningioma, diagnostic delay, examiner bias, focal neurological deficit, foot drop, gait instability, neuro-monitoring, proprioceptive loss, vasogenic brain edema

## Abstract

Meningiomas are common intracranial tumors, but their slowly progressive and atypical presentations are frequently mistaken for peripheral neurologic, musculoskeletal, or ocular conditions. We describe a 60-year-old left-handed man who developed several months of progressive right-sided gait imbalance, proprioceptive deficits, intermittent binocular visual blurring, and late cognitive changes. Evaluations by podiatry, chiropractic care, physical therapy, and optometry initially failed to uncover the cause. Brain MRI eventually revealed a large left posterior parietal dural-based mass with classic meningioma features, marked vasogenic edema, mass effect, and midline shift. Gross-total resection confirmed a WHO grade 1 meningioma (Ki-67 index 15 %). This case emphasizes an atypical presentation with progressive unilateral gait dysfunction accompanied by proprioceptive loss, pyramidal signs, visual symptoms, or cognitive decline. Refractory treatments should prompt early neuroimaging, even after negative peripheral workups. It also highlights how specialty-specific anchoring bias can delay recognition of central nervous system lesions.

## Introduction

Meningiomas are the most common primary intracranial tumors in adults and demonstrate a clear female predominance with peak incidence in the elderly population according to Central Brain Tumor Registry of the United States (CBTRUS) data [1]. Although most symptomatic meningiomas present with headache and seizures, this case is distinctive for its insidious, lower-extremity-predominant proprioceptive gait imbalance and foot-drop-like symptoms that closely mimicked isolated peripheral foot pathology for months in a male patient, despite a large tumor with marked vasogenic edema and midline shift. Meningiomas are typically slow-growing and extra-axial; clinical presentation varies widely depending on location, size, and surrounding edema [2]. The parietal cortex plays a central role in integrating somatosensory input, proprioception, and spatial awareness of the contralateral body. Consequently, a parietal convexity meningioma can plausibly give rise to focal sensory disturbances or altered limb-position perception, symptoms that may readily be mistaken for peripheral nerve or musculoskeletal pathology. This clinical overlap may encourage anchoring on an initial non-neurologic diagnosis. As a result, this can delay reconsideration of an intracranial cause when symptoms persist or progress. We report a large left posterior parietal meningioma that presented in precisely this manner, illustrating how atypical focal sensorimotor symptoms, specialty-specific anchoring bias, and fragmented care can postpone diagnosis of an intracranial mass.

## Case presentation

A 60-year-old left-handed man with no significant past medical history presented with several months of progressive right-sided gait imbalance. Further questioning revealed proprioceptive deficits, intermittent binocular visual blurring, and recent cognitive changes. He had been living independently and driving until his family noticed worsening coordination.

Approximately four months earlier, he first noticed subtle right-foot instability, especially during walking or simple tasks such as putting on shoes and frequent tripping over door thresholds. Because the symptoms initially appeared focal to the right foot rather than involving the entire leg or multiple neurologic domains, concern for a central neurologic process was not raised during the early referrals. Podiatry evaluation, including blood work, foot radiographs, and foot MRI, was unremarkable. Persistent symptoms led him to chiropractic care, neurologic chiropractic therapy, and physical therapy, none of which halted progression. He then developed intermittent blurry vision (binocular, without diplopia or field defects on bedside testing). Optometry confirmed only a pre-existing refractive error with no known fundoscopy; therefore, the presence or absence of papilledema could not be confirmed. New lenses brought no relief. Cognitive symptoms were not present early in the course. In the final two weeks before presentation, family members noticed slowed responses and occasional disorientation, and the patient reported needing to think harder to retrieve common information, such as when performing simple math. Neurology was not involved before the emergency department evaluation. The timeline of his evaluations across multiple specialties prior to diagnosis is summarized in Table 1.

**Table 1:** Diagnostic timeline and pre-diagnostic specialty evaluation.

Time point	Specialty/setting	Workup or intervention	Outcome
Initial evaluation, ~four months before ED visit	Primary care	Evaluated subtle right-foot instability, blood work, and X-ray of the foot	Symptoms were initially approached as a focal foot or musculoskeletal problem
~Four months before ED visit	Podiatry	Further foot radiographs (MRI) for right-foot instability and physical exam testing.	Workup unremarkable; symptoms continued
~ Three months before ED visit	Chiropractic care/neurologic chiropractic therapy	Conservative therapy for persistent gait/foot symptoms	No improvement; symptoms progressed
~ Two months before ED visit	Physical therapy	Gait and mobility-focused therapy	Symptoms persisted despite treatment
~ Three weeks before ED visit	Optometry	Evaluation for intermittent binocular blurry vision; new lenses prescribed	Only pre-existing refractive error identified; no symptom relief
Final two weeks before ED visit	Home/family observation	Worsening coordination, slowed responses, occasional disorientation, difficulty with simple math	Emergent evaluation prompted
ED evaluation	Emergency/neurologic evaluation	Neurologic examination and brain MRI	Large left posterior parietal dural-based mass identified
After diagnosis	Neurosurgery	Left craniotomy with neuronavigation	WHO grade 1 meningioma confirmed; postoperative improvement of symptoms

He denied headache, seizures, loss of consciousness, diplopia, dysarthria, dysphagia, chest pain, or shortness of breath. On examination, he was alert but fatigued. Neurologic findings included right-sided hemiparesis (worse in the leg) with subtle foot slapping on ambulation, brisker right-sided reflexes, an extensor plantar response, impaired proprioception and light touch in the right lower limb, and a positive Romberg sign. Gait was broad-based and unsteady. The neurological examination was negative for hemispatial neglect, agraphesthesia, and astereognosis. 

Brain MRI demonstrated a large (~7 cm) avidly enhancing dural-based mass arising from the left parietal dura with a dural tail, partial calcification, and extensive vasogenic edema causing rightward midline shift, mass effect, and effacement of the left lateral ventricle-features consistent with meningioma (Figures 1, 2). No acute infarction or hemorrhage was present.

**Figure 1:**
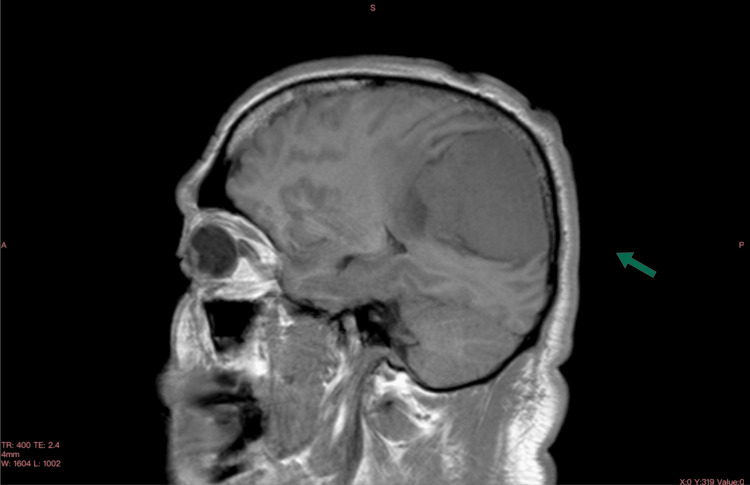
Sagittal T1-weighted MRI demonstrating a large approximately 7 cm, avidly enhancing dural-based mass. In the left posterior parietal region with a classic dural tail sign, a significant mass effect and a rightward midline shift can be visualized.

**Figure 2:**
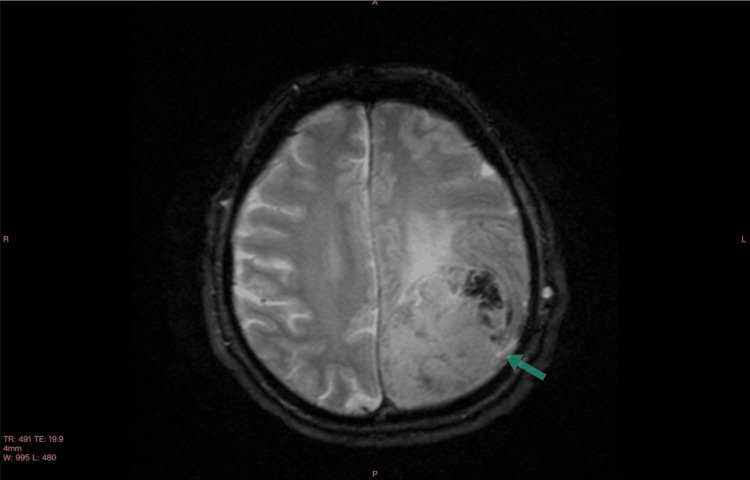
Axial T2-weighted MRI showing the heterogeneous approximately 7 cm left parietal mass with prominent surrounding vasogenic edema, midline shift, and effacement of the left lateral ventricle.

The patient underwent left craniotomy with neuronavigation. Pathology confirmed a WHO grade 1 meningioma (Ki-67 proliferation index of 15%, no atypical or anaplastic features). He tolerated surgery well and showed gradual improvement in gait stability and proprioception postoperatively. Adjuvant radiation was administered, followed by serial MRI surveillance every six months; the cavity has remained stable with no recurrence.

## Discussion

This case illustrates a diagnostically challenging presentation of a large left parietal convexity meningioma. The dominant early symptom was an insidious, progressive right-sided proprioceptive deficit and gait imbalance that closely mimicked a peripheral podiatric or musculoskeletal problem. Despite the tumor’s substantial size, marked vasogenic edema, midline shift, and ventricular effacement, classic meningioma symptoms such as headache, seizures, and overt raised intracranial pressure were absent. The discrepancy between impressive imaging findings and relatively subtle, lower-extremity-predominant deficits highlights how slow-growing extra-axial lesions can take advantage of cerebral plasticity and compensatory mechanisms, leading to a delay in decompensation.

Neuroanatomically, the large left posterior parietal meningioma primarily affected the medial portion of the contralateral (right) sensorimotor cortex. Within the somatosensory and motor homunculus, the lower extremity and foot are represented most medially in the paracentral lobule, adjacent to the midline [3]. This landscaped organization explains the clinical predominance of right leg proprioceptive loss, gait imbalance, and mild foot drop, with relative sparing of the more lateral arm and face regions. Furthermore, the tumor’s slow growth permitted extensive cerebral plasticity and compensatory mechanisms-such as synaptic reorganization, recruitment of adjacent cortical areas, and utilization of redundant neural pathways-to mitigate the effects of progressive mass effect and vasogenic edema [4]. This adaptation accounts for the notable discrepancy between the impressive radiographic findings (large tumor size, significant edema, and midline shift) and the relatively subtle, insidious clinical presentation.

Pathologic analysis confirmed a WHO grade 1 meningioma. Histologic examination demonstrated nuclear crowding, foci of necrosis, and small cell change; however, mitoses were infrequent on PHH3 staining. Immunohistochemical stains for SSTR2A, EMA, and PR were positive within tumor cells, and the Ki-67 proliferation index was focally elevated at up to 15%. Molecular testing for TERT promoter mutation was negative, chromosomal microarray analysis was negative for CDKN2A/B homozygous deletion, and methylation profiling matched the meningioma class (DKFZ v11b4). While the Ki-67 index exceeds the typical range reported for benign meningiomas (<5-10%) [5], the absence of definitive atypical or anaplastic features sufficient to meet WHO criteria for a higher grade, together with negative high-risk molecular alterations, preserved the grade 1 designation. The elevated Ki-67 nevertheless indicates increased proliferative potential and has been independently linked to higher recurrence risk even in grade 1 tumors [5]. Although adjuvant radiotherapy is not routinely recommended after gross-total resection of WHO grade 1 meningiomas [6], the combination of this markedly elevated proliferative index, large tumor size, and significant mass effect with midline shift prompted a multidisciplinary team decision to administer adjuvant radiation followed by serial MRI surveillance. The patient’s rapid postoperative recovery with no recurrence underscores the benefit of timely intervention once the correct diagnosis is established.

Atypical presentation and literature gap

According to a recent narrative review aggregating data from heterogeneous studies [7], intracranial meningiomas most often present with headache (33-37% of symptomatic cases), seizures (17-25%), cognitive or personality changes (~14%), or focal motor/sensory deficits (10-30%). Gait imbalance or ataxia occurs in only about 6% overall [7]. Ataxia and imbalance are far more common in posterior-fossa or parasagittal lesions than an isolated, predominantly proprioceptive gait disturbance from a convexity parietal meningioma [8].

While parietal and convexity meningiomas can cause contralateral hemiparesis, the specific phenotype seen here-an insidious, lower-extremity-predominant proprioceptive gait issue, attributed to foot pathology-is infrequently highlighted in the literature and represents an under-recognized variant [9-13].

The four-month diagnostic journey in this patient (podiatry with normal imaging, chiropractic/neuro-chiropractic care, physical therapy, and optometry) exemplifies a gap in the literature regarding central nervous system lesions presenting as peripheral complaints. Limited literature reports detail the multi-specialty referrals that occur when focal neurologic signs are narrowly viewed. This case provides evidence that these types of delays carry consequences: repeated ineffective treatments, increased healthcare costs, prolonged functional impairment, and the risk of sudden deterioration.

Specialty silos and cognitive biases

Anchoring bias and specialty-specific silos played a central role in the diagnostic delay. Each provider appropriately focused within their domain, podiatry on foot mechanics, chiropractic on spinal alignment, optometry on refractive error, yet did not pivot when symptoms proved refractory. Cognitive-bias research shows that specialists are especially prone to anchoring on an initial “peripheral” hypothesis when classic intracranial red flags are absent [14]. These cognitive errors were compounded by systemic barriers to timely neurological care. Significant neurologist workforce shortages across the United States have resulted in prolonged wait times for specialty evaluation, particularly in underserved and vulnerable communities [15]. Had this patient been referred to neurology earlier in the course, neuroimaging and definitive diagnosis could likely have been obtained weeks to months sooner, potentially limiting functional decline and improving clinical outcome. This case underscores how shortages in medical and surgical specialties can exacerbate diagnostic delays and disproportionately affect vulnerable populations.

## Conclusions

Large parietal meningiomas can present with progressive unilateral gait imbalance and proprioceptive loss that closely mimics peripheral foot pathology. This case shows how failing to recognize this atypical pattern, combined with anchoring bias, specialty silos, and fragmented care, leads to significant diagnostic delay. Progressive unilateral proprioceptive or gait disturbances that fail to improve promptly with targeted peripheral therapy deserve brain MRI, even without headache or seizure. Clinicians should maintain a broad differential that includes intracranial mass lesions whenever symptoms persist or cross specialties. Early neuroimaging in atypical or refractory focal neurologic complaints can prevent prolonged morbidity and avert catastrophic decompensation.

This case adds to a small but growing body of literature describing intracranial lesions that may initially mimic peripheral complaints. It highlights specific red flags that should prompt reconsideration of a central nervous system cause, including progression despite targeted peripheral therapy, upper motor neuron signs, binocular visual symptoms, and late cognitive changes. Early neurology involvement would likely have avoided much of the four-month diagnostic delay, as recognition of these features would have prompted urgent brain and spine imaging.
